# Educating the energy informatics specialist: opportunities and challenges in light of research and industrial trends

**DOI:** 10.1007/s42452-021-04610-8

**Published:** 2021-05-30

**Authors:** Chiara Bordin, Sambeet Mishra, Amir Safari, Frank Eliassen

**Affiliations:** 1 UiT, The Arctic University of Norway, Tromsö, Norway; 2 Tallinn University of Technology, Tallinn, Estonia; 3 Danish Technical University, Kongens Lyngby, Denmark; 4 Euro Energy Solutions AS, Oslo, Norway; 5 University of South-Eastern Norway, Kongsberg, Norway; 6 University of Oslo, Oslo, Norway

**Keywords:** Energy informatics, Mathematical optimization, Renewable energy, Sustainability, Education, Industrial innovation, Interdisciplinary

## Abstract

**Abstract:**

Contemporary energy research is becoming more interdisciplinary through the involvement of technical, economic, and social aspects that must be addressed simultaneously. Within such interdisciplinary energy research, the novel domain of energy informatics plays an important role, as it involves different disciplines addressing the socio-techno-economic challenges of sustainable energy and power systems in a holistic manner. The objective of this paper is to draw an overview of the novel domain of energy informatics by addressing the educational opportunities as well as related challenges in light of current trends and the future direction of research and industrial innovation. In this study we discuss the energy informatics domain in a way that goes beyond a purely scientific research perspective. This paper widens the analyses by including reflections on current and future didactic approaches with industrial innovation and research as a background. This paper provides key recommendations for the content of a foundational introductory energy informatics course, as well as suggestions on distinguishing features to be addressed through more specialized courses in the field. The importance of this work is based on the need for better guidelines for a more appropriate education of a new generation of experts who can take on the novel interdisciplinary challenges present in future integrated, sustainable energy systems.

**Article highlights:**

- Didactic approaches in the energy informatics domain are discussed based on research and industrial trends.
- Research trends and industrial innovation driven by energy informatics are investigated.
- A fundamental framework for an energy informatics course is defined together with specialized distinguishing features.

## Introduction

The energy sector is facing increasing challenges and is undergoing significant changes that increase its complexity and have led to the need for innovative energy management strategies. Currently, 85% of the world population utilizes electricity, with coal representing the main resource for 40% of the world’s electricity [1]. Coal also produces 70% of the carbon dioxide emissions (CO2) from the generation of electricity. The continuing electrification of the world, which is driven by increasing world energy consumption, is adding pressure to the overall system. This is evidenced by the fact that in 2018 energy demand worldwide grew by 2.3%, which is nearly twice the average rate of growth since 2010. The demand for all fuels increased, with fossil fuels making up nearly 70% of that growth. As a result, global energy-related CO2 emissions rose by 1.7% to 33 Gigatonnes (Gt) in 2018 [2, 3].

The EU has set long-term goals of reducing greenhouse gas emissions by 80–95% when compared to 1990 levels by the year 2050 [4]. With the so-called “European Green Deal,” the EU aims to be climate-neutral by 2050. Climate neutral means an economy with net-zero greenhouse gas emissions, which will create the need for decarbonisation of the energy system while at the same time ensuring the security and safety of energy supply.

Moreover, in September 2015, the United Nations General Assembly formally adopted the 2030 Agenda for Sustainable Development. This comprises a set of 17 Sustainable Development Goals (SDGs) [5] to address the global challenges the world is facing, including those related to poverty, inequality, climate change, environmental degradation, peace and justice [6]. Among these goals, there are a few that are deeply connected to challenges in the energy sector. These include ensuring access to affordable, reliable, sustainable and modern energy (SDG 7); building resilient infrastructure, promoting sustainable industrialization and fostering innovation (SDG 9); making cities inclusive, safe, resilient and sustainable (SDG 11); ensuring sustainable consumption and production patterns (SDG 12); and finally, taking urgent action to combat climate change and its impacts (SDG 13).

Environmental, economic, and social sustainability therefore represent three main dimensions for creating a more environmentally friendly society that encompasses the SDGs [7].

## Motivation: interdisciplinary needs in energy research

### Socio-techno-economic perspectives in energy

It is evident that sustainable production and consumption together with renewable electrical energy systems are major concerns around the world. These concerns involve technical, economic and social aspects that must be addressed holistically in order to properly achieve the long-term goals of the EU as well as the broader Sustainable Development Goals.

Broadly speaking, technical aspects refer to power quality [8], power system reliability [9, 10], (with regard to system adequacy, security, and stability), predictive maintenance and monitoring systems [11]. Economic aspects refer to investment decisions (especially regarding renewable resources and storage technologies), optimal operational management of energy sources for costs minimisation, electricity markets [12], novel trading mechanisms between microgrids [13, 14], and technological learning curves [15]. Social aspects are also playing an increasingly key role within energy systems as social science research shows how “humans energy behaviour” [16, 17] can affect power and energy systems, both technically and economically. Demand response [18], arbitrage [19], and the wider and more modern concept of “nudging” [20, 21] are all means through which it is possible to impact human behaviour and motivate final energy users towards more sustainable choices. Such sustainable choices will be put into effect in the form of specific investment and operational decisions, which will in turn impact the overall energy and power systems’ economic and technical aspects.

### From multidisciplinary to interdisciplinary research in energy

The holistic socio-techno-economic perspectives outlined above require highly interdisciplinary approaches in order to properly tackle the various issues of power and energy systems.

Indeed, “interdisciplinarity” is currently a buzzword in contemporary energy research. Intuitively, it refers to some theoretical or methodological “bridging” between disciplines. This enables disciplinary “imports” and “exports” [22]. Authors in [22] and [23] highlight how interdisciplinarity is differentiated from the more common multidisciplinary approach. The latter emphasises the collaboration between several disciplines around a common research topic in a way that is additive rather than integrative. The multidisciplinary approach focuses only around a single object while somehow remaining compartmentalised and juxtaposed. An interdisciplinary approach, on the other hand, is a co-construction of the research process that aims at integrating the perspectives of different disciplines in order to achieve a synthesis of knowledge, as well as provide a holistic understanding of the problem [24]. Interdisciplinarity is therefore the paradigmatic change that is needed in energy research in order to address today’s complex world and to achieve the wide variety of energy related goals. Indeed, a multitude of challenges are posed by the transition of energy systems towards sustainability. Most of these challenges are social-technical-economic in nature and so therefore require interdisciplinary collaboration [25].

### Energy informatics: a key for interdisciplinary energy research

Within the interdisciplinary approach outlined above, the novel domain of energy informatics plays an important role: it involves different disciplines in addressing the socio-techno-economic challenges of energy and power systems holistically. In particular, the goal of energy informatics is to use emerging new information and communication technologies to make energy systems more efficient, effective, safe, secure, economical and clean. Energy informatics utilizes digital technology and information management theory and practice to facilitate the global transition towards sustainable and resilient energy systems [26]. The authors of [27] define energy informatics as a subject in which researchers aim at “exploring the intersection of informatics, power engineering and energy economics”. The two main goals of energy informatics can be identified as “energy efficiency” and “renewable energy supply” [28]. In short, the scientific literature portrays energy informatics as a field that includes topics such as smart (power) grids, renewable systems, smart meters, demand response, smart buildings, electric mobility, energy storage, data centres, energy policy, energy markets and market mechanisms.

## Objective, key contributions and structure of the paper

The objective of this paper is to draw an overview of the novel domain of energy informatics by addressing the educational opportunities as well as related challenges in light of current trends and the future direction of research and industrial innovation.

The key contribution of this study is to look at the novel energy informatics domain in a way that goes beyond the purely scientific research perspective (which usually happens in the literature). Compared to the available literature in energy informatics, this paper widens the analyses by including reflections on current and future didactic approaches, with industrial innovation and research as a background.

The main motivation for expanding this analysis is that research and industrial needs should drive education choices. It is commonly agreed that teaching at the university level should be research-based; therefore, it would be ill advised to focus on discussing teaching approaches without introducing the overall current status of research in the field. Proposed teaching methods should have an eye on the most popular research trends. Similarly, proposed teaching approaches should not be disconnected from industrial innovation trends, as universities should teach not only for those who will continue their path in the academic research, but also for those who will work in the real industrial world.

**Fig. 1:**
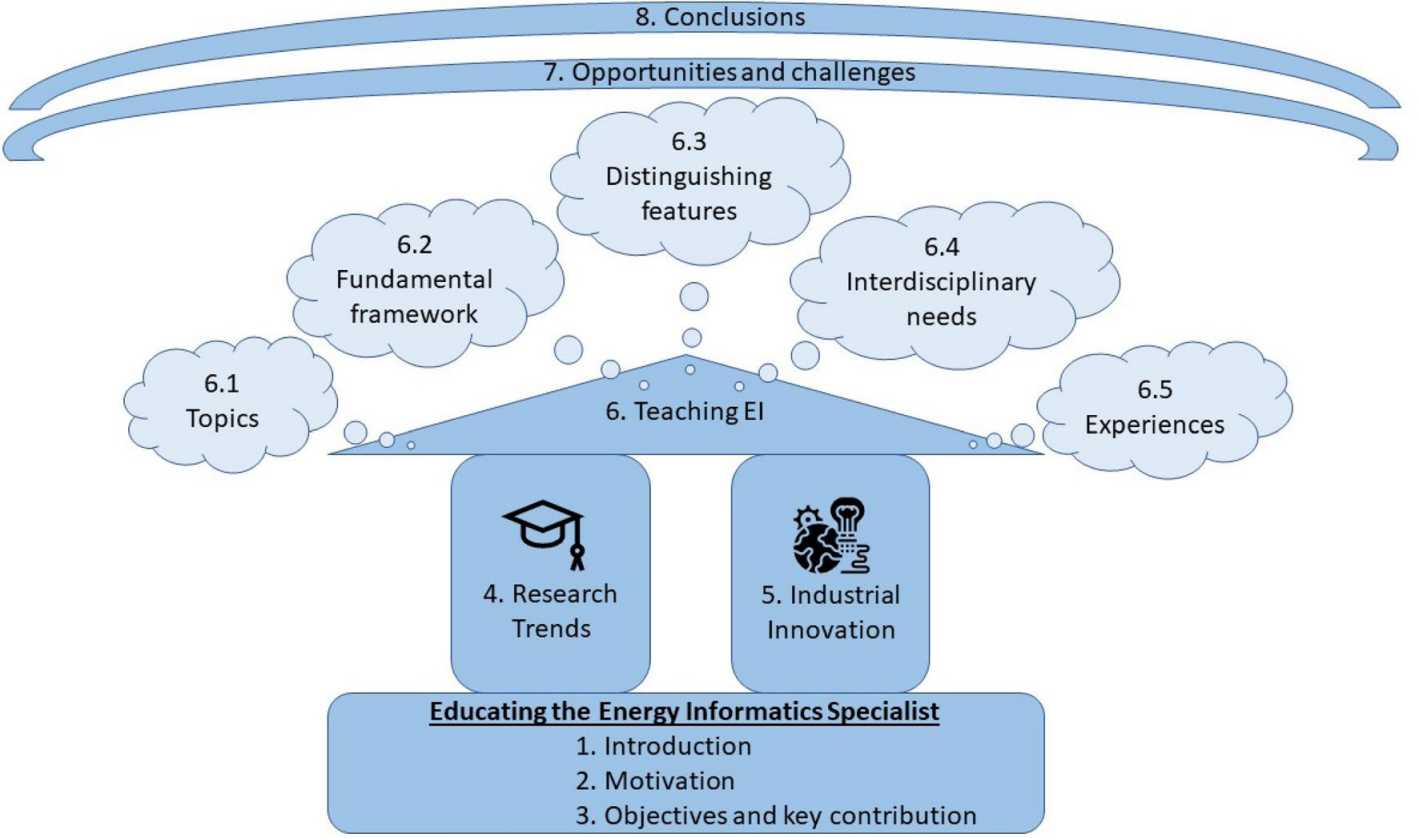
Structure of the paper. Sections and subsections ordered from the bottom to the top

The structure of this paper is summarized in Fig. 1. The Sects. 1, 2, and 3 present an introduction, a motivation for the proposed work, the objective and key contributions respectively. They therefore lay the foundation of the work and represent the base of Fig. 1. Afterwards, three main sections are proposed to reflect on the three main drivers of research, industrial innovation, and education. Section 4 will provide a broad overview of the current research trends in energy informatics in order to present the big picture from a high level perspective. This section is not supposed to be exhaustive, but rather is aimed at introducing the overall context from the research perspective, and broadly touches on the main topics that are addressed within the energy informatics domain. Afterwards Sect. 5 will outline the main concepts linked to industrial innovation in the field such that the reader will be given hints on current industrial trends. The following Sect. 6 will discuss how the novel domain of energy informatics can be better communicated to young students, the main topics that should be addressed within energy informatics courses, a proposal for a fundamental framework for the course, as well as suggestions on distinguishing features to be addressed through more specialized courses in the field.. The section also discusses how to address the interdisciplinary needs of an energy informatics course, as well as real-world experiences of past and current energy informatics and master level courses. Figure 1 shows how Sects. 4 and 5 represent the pillars on which Sect. 6 is built. It is clear that education choices discussed in Sect. 6 should be driven by the research and industrial needs discussed in Sects. 4 and 5. Figure 1 shows also the subsections included in Sect. 6, for completeness (included within the light blue clouds). Opportunities and challenges of interdisciplinary research teams will be discussed in Sect. 7, based on the reflections made earlier. Conclusions and recommendations will be drawn in Sect. 8 in light of the main topics discussed in the previous sections.

## Research trends in energy informatics

Energy informatics is a field that sits at the intersection of physics, chemistry, engineering, management and sociology with energy as the focus. Expanding further, power and energy systems, computer science and social sciences are the major directions. Energy can be understood as the power resulting from any physical or chemical processes with the objective to obtain light, heat and mechanical work.

Energy informatics formally combines the streams of computer science that are applied to energy and environmental problems. The field can be broadly classified as based on the level of energy content, specifically the ratio between the energy-oriented focus to the informatics-oriented focus. This ratio largely depends on the premise of the research. For example, the Internet of Things (IoT) with a focus on energy losses and power system control for large wind parks, falls under the scope of energy informatics. In sum, by definition, the scope of energy informatics is wide with a focus on energy management as a cross cutting theme.

Table 1 outlines fields and topics trending in energy informatics research. This is a summary of the main topics that were found while reviewing the available literature. After studying the literature, it is possible to summarize and classify the relevant works as they are shown in the Table 1.

**Table 1:** Research directions

Field	Topics
Data science	Big data, small data, statistics, data engineering and data architecture
Sustainable power and energy systems	Electrical and electronics engineering, power system, energy system, power system digitalization, energy markets, peer-to-peer trading, renewable sources integration
Computer science	Green computing, algorithms and operating system, cyber-physical systems, Internet of Things, interfaces, cyber security, edge/fog/cloud computing
Economics	Macro and micro economics
Mathematics	Operations research
Social factors in energy systems	Energy users behaviour, nudging for sustainable energy choices
Law and regulations	Rights and responsibilities of the power consumers, frameworks for better management of energy

The term “energy informatics” was first introduced in [29]. The authors discuss how the information systems community could contribute to environmentally sustainable development. In [28] the authors point to the challenges and opportunities for computer science and information systems to move towards an efficient and integrated power grid. In the process of integrating decentralized and distributed energy systems, standard communication protocols are a pre-requisite. Increasingly, the technology is moving towards an integrated and communicative system. In [30] the authors expand on communication protocol standardization in a smart grid architecture. Note that the standardization of protocols often requires experts in different disciplines such as communications, electronics systems, and computer systems. Energy informatics can act as a bridge in organizing such complex and multi-disciplinary subjects.

Data from sensors and internet connected things is readily available in large volumes. The need to extract meaningful information from this data has lead to a recent surge in the data analysis sector. In the context of energy, apart from obvious demand and generation data, there are more complex data sets becoming available, such as stored data (data-at-rest) and streaming data (data-in-motion). In [31, 32] the authors refer to the big volume of energy related data and how machine learning applications can be adapted to tackle this big data. Energy Informatics can leverage the advances made available in computing to address complex and real-world energy issues.

The power system is becoming more decentralized and distributed. Renewable energy resources are also increasing their share of the total energy portfolio. In [33] the authors refer to end-user-based demand side management in a multi-microgrid environment. The authors in [34] expand on power electronic interfaces in a distributed generation. In [35] the authors study the energy aware data platforms. With the emergance of local and distributed generation, energy informatics facilitates new and innovative business prospects along with management of complex networked systems.

The transportation sector is witnessing a rise in electric vehicles (EV). Not only are they environmentally friendly, but they can also act as a means for storing energy when the prices are low and the discharge when high. In [36] the authors elaborate on smart charging of electric vehicles and vehicle-to-grid (V2G) operations. In [37] an energy aware traffic control mechanism is studied. In [38] models for optimal charging sites design, expansion, and operations are proposed to investigate the trade-offs between network reinforcement and storage integration, with multi-horizon perspectives. A real world, data-driven, decision support system for electric vehicle charging infrastructure development is discussed in [39].

Habitations and household equipment are becoming smarter [40]. This has opened up new opportunities and challenges with coordination for optimal and efficient use of energy [41]. Smart energy meters are being deployed on a large scale in the EU [42]. In [43] the authors discuss smart buildings and smart grid features in the context of an energy city. In [44] the authors explore the potential of IoT and energy management for a residential building. An innovative building management system for automated auditing and continuous building commissioning is proposed in [45].

In the domain of social sciences, consumer behavior is investigated in order to better understand the potential of demand side flexibility [46]. In [47] the authors discuss the role of nudging, or guidance, for energy efficiency aware decision making for consumers. This sheds light onto research questions related to behavior change, occupant comfort and user interactions. In [48] the authors investigate coordinated energy management for occupant comfort using a multi-agent system. In [49] the authors present consumer interaction, considering different smart grid projects in the EU. Currently the power system, from generation to the consumer level, is moving towards tight integration and coordination. Energy informatics can bring together experts from different fields to achieve a holistic and optimal solution to this coordination.

Privacy challenges are becoming bigger with the increasing rise in the integration of energy systems. In [50] the authors highlight the privacy issues concerning smart energy meters with a case study from the Netherlands. In [51] the authors study the connectivity verification of distribution networks through data analysis by considering smart energy meters. In [52] the authors expand on the cyber threats and security concerns in the smart grids in Europe. The energy laws need adjustments to keep up with the pace of advancements in technologies. In [53] the authors shed light on current and existing energy laws in Europe.

Research is becoming more interdisciplinary as the developments in information and communication technologies (ICT) brings us ever more closer and integrated. The current trends demonstrate that there is a need for standardization in order to streamline research. Energy informatics is an interdisciplinary approach that brings forth state-of-the-art of computer science and electrical engineering. Energy informatics can facilitate a convergence of advancements in computer science applied to real-world power and energy system issues while also considering the societal aspects. Current research leans towards multi-disciplinary approaches with experts from different fields. In contrast, energy informatics proposes an interdisciplinary approach, thereby synthesizing the knowledge and methods from different disciplines. The law of geography says that everything is in relation to everything else, while the local regions are more so. Thereby there is no “one solution fits all”. For instance, a solution for northen Europe is not applicable to the Baltics and vice-versa. While the fundamentals of energy informatics remain the same, the solutions need to be adapted to the geographical constraints of each region.

### Renewable energy systems and sustainability: a common bridge between disciplines

Renewable energy integration and sustainability are the main motivations for the need to link the different disciplines outlined in the previous section under the umbrella of energy informatics. Renewable energy supply has been identified among the main scope of energy informatics research, together with smart grids, energy efficiency, and smart energy saving systems [28].

Environmental, economic, and societal sustainability can be achieved thanks to the interdisciplinary approach provided by energy informatics [54] because ICT allows environmental and energy efficiency issues to be handled effectively [55]. Indeed, energy informatics offers promising opportunities for energy and utilities companies to experiment with new business models for addressing energy efficiency and climate-protection goals, and thus encourages their customers to save energy [56]. From this point of view, the concept of “eco-cities” arises as the most environmentally sound model of sustainable urbanism. Authors in [57] highlight that the eco-city needs to embrace and leverage the advanced ICT opportunities, particularly with regard to sustainable energy systems, so as to improve its contribution to the goals of environmental sustainability. In this context, energy informatics provides exactly the interdisciplinary and holistic approach to tackle such goals.

To conclude, the intrinsic interdisciplinary aspects of the energy informatics domain outlined so far is well reflected in the increasing complexity that the energy and power industries have to face nowadays, especially when they deal with renewable and sustainable systems. In a world that is becoming more and more interconnected, the energy and power industries must cover different needs holistically while a new nexus between sectors arises. In order to succeed and become more sustainable, the industry can no longer limit itself to one or a few objectives strictly related to production, but they must address problems at social, economic, technical, and legislative levels together. This is where both research and education must come into the picture to create trained experts who can take on the new interdisciplinary challenges of the industrial world and move towards greener solutions. At the same time, the links between education, research and industry in the field of sustainable energy and power systems should be strengthened. On the one hand, the research tasks can be driven by industrial needs, and on the other hand, industry needs can evolve based on new inputs coming from fundamental research.

## Industrial innovation driven by energy informatics

Universities need a kind of partnership with industry and/or business people to advance the educational development within novel fields of engineering. This partnership with industry can contribute to these fields both by providing advice on program development and by giving innovation-oriented lectures in courses to allow students to achieve higher levels of hands-on knowledge. Such a proactive collaboration will be more successful in producing the kind of capable graduates the market and industry seek to satisfy all the economic, technical, and legal needs of sustainable development.

Energy informatics, which is an interdisciplinary area merging several new promising technologies, especially requires a creative model of the relationship between academia and innovation. This interdisciplinarity has to be introduced in an optimal framework where both research and training will be implemented. On one hand, it can be useful to employ an industry advisory group to ensure that educational plans are relevant to current and future industrial demands. On the other hand, a scientific committee should examine the accuracy and relevancy of the educational model for graduate students in energy informatics.

A useful tool for launching scientific disciplines in the field of energy informatics at universities, in accordance with the challenging needs of the industrial sector, would be using the “hype cycle method”. It gives an overall view of how energy informatics technologies are projected to advance over a specific period of time. This tool also introduces university professors and their graduate students to the current status of innovation in this field and encourages them to research existing and future challenges as dissertations or in graduate courses.

The hype cycle that was developed and is being used by the American research, advisory, and information technology firm, Gartner [58], represents the maturity, adoption, and social application of specific technologies. Each hype cycle is divided into the five key phases of a technology’s life cycle as follows: (1) Innovation Trigger in which early proof-of-concept stories and media interest trigger substantial promotion; (2) Peak of Inflated Expectations where early publicity produces a number of success stories and only some companies take action; (3) Trough of Disillusionment where fondness declines as tests/implementations fail to deliver so that investment continues only if the remaining providers modify the products; (4) Slope of Enlightenment in which second- and third-generation products appear from technology providers and more enterprises supply pilots; and (5) Plateau of Productivity (not shown in the figure) where mainstream adoption jumps, and if the technology has more than a niche market then it will continue to grow [58].

**Fig. 2:**
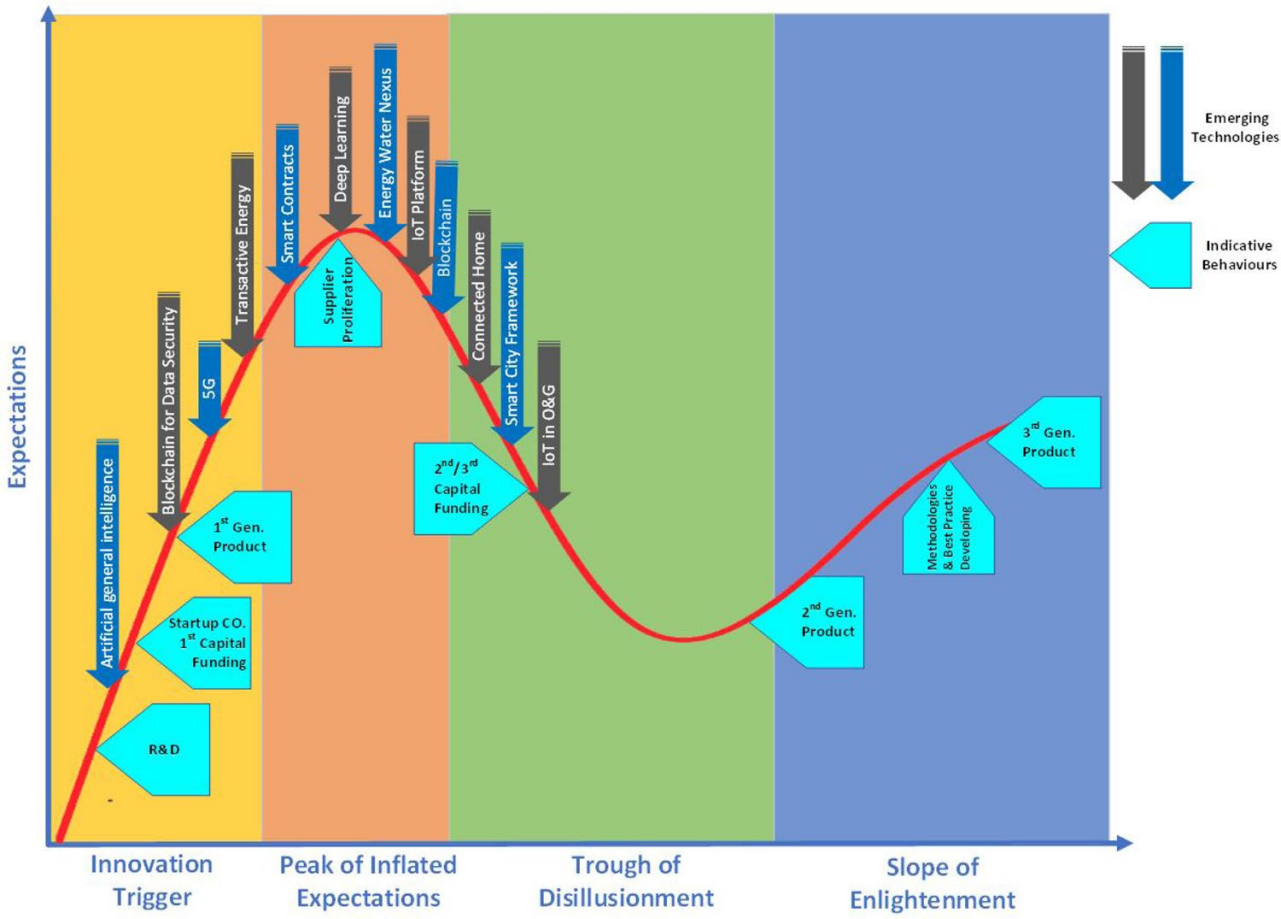
Emerging technologies hype cycle—inspired by [59] and adapted for the energy informatics specific case

Figure 2 is inspired by [59] and has been adapted by the authors to discuss the specific case of the energy informatics domain. As shown in the figure, blockchain for data security [60], artificial intelligence (AI) [61], 5G [62], and transactive energy [63] are in the technology trigger phase where some proof-of-concept stories and media interest leads to popularity. The plateau will be reached in 2–5 years for the blue and in 5–10 years for the black vertical arrows.

At the same time, smart contracts, deep learning, IoT platforms, energy water nexus, and blockchain in general are in the peak of inflated expectations in which some companies endeavor to show a number of success stories in reality. The smart city and connected homes/cars are examples for industrial application of energy informatics concepts, which are located in the trough of disillusionment phase and rapidly growing with the increased use of renewable sources, onsite generation, ICT, and distributed control. On the one hand conventional grids address centralized power generation, little renewable power generation, limited grid access for new producers, one-directional power flow, and operation based on experience. On the other hand, smart (micro)grids respond to centralized and distributed power generation, significant (onsite) renewable power generation, prosumers, multi-directional power flow, and operation based more on real-time data. Conventional grids have proven reliable, but they have challenges with increased intermittent renewable integration, decentralization and efficiency. This means that there is considerable potential here to change into a more reliable, robust, and economic electricity grid for the future of the energy world.

Indicative behaviors of different phases in the hype cycle have been also shown in Fig. 2 where, for example, R&D that was discussed in the previous section appears in the beginning of the technology trigger phase. After that, and in the same phase, startup companies come in for the first round of venture capital funding to realize the first generation and/or minimum viable product (MVP). While both mass media and negative press will begin in the next phase, second/third rounds of venture capital funding would happen in the third phase (i.e., trough of disillusionment). In the fourth phase, however, methodologies and best practices, as well as third-generation products, finally develop.

**Fig. 3:**
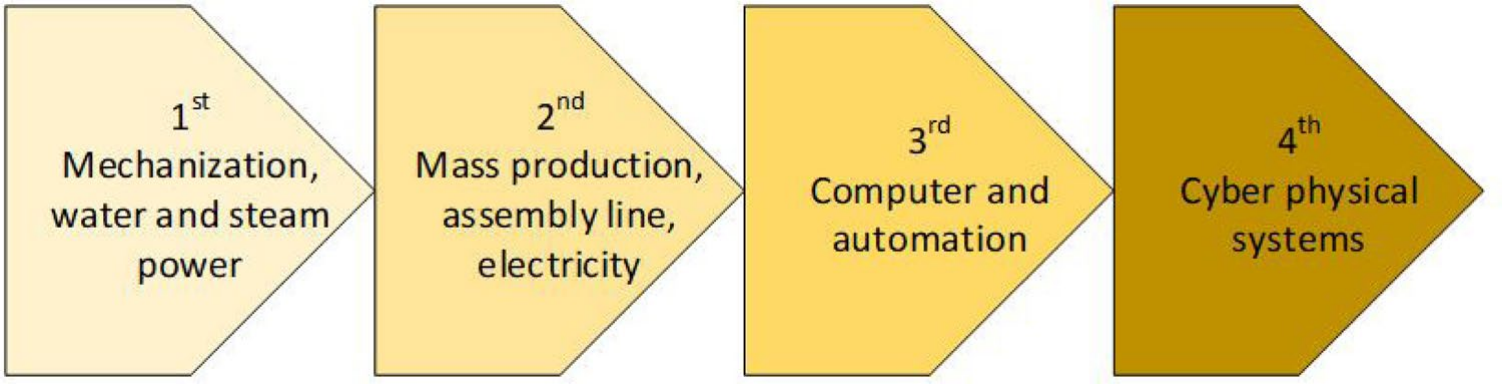
Industry 4.0 trend

Energy informatics and its applications can be considered cyber-physical systems, and therefore examples of the fourth industrial revolution (Fig. 3). The fast advances in ICT have changed our perception of the real world into a perception of virtual things and Internet of Everything [64]. Many stakeholders around the world have leveraged these advances to mitigate different challenges in the environmental and energy sectors. In this way, both developed and developing countries can benefit from ICT to expose the issues concerning climate change, waste management, energy poverty, and energy efficiency [65]. For instance, ICT would play a major role in initiating and enabling the EU to reach its energy efficiency targets, for example on building performance, smart meters, and demand response, thanks to the ability for advanced measuring, monitoring, and control [66].

One important example is an integrated building energy management system (iBEMS), which is defined as a comprehensive platform that facilitates management and control of energy production, saving, and consumption. This facilitation is for a community of smart buildings where distributed energy prosumers, energy industries/companies, and local authorities could be the target groups of such a system. Buildings will play a critical role not only in energy efficiency but also in a wider changing energy grid system [66]. In terms of residential application, TrendForce cross-examination of upcoming developments in the energy and communication sectors provides some insight into what technologies may push the residential/commercial sector forward in 2019 and beyond [67]. The main focus may be on ICT, and there is still substantial crossover with energy sectors that shape the future use of technologies like commercial 5G, smartphones, memories, mini LED, eSIM, advanced sensors, EMS, etc. and their trends in the home for the years ahead in both energy generation and energy consumption.

There is one important final point to make concerning new business models affected by the energy informatics concept. Energy service companies (ESCOs) find themselves in a paradoxical situation in which their traditional business models are losing profitability when they have to consider energy efficiency and climate-protection goals, and thus encourage their customers to save energy [56]. From this perspective, three types of technology can create an intelligent energy-management system including flow networks, sensor networks, and sensitized objects [29, 56].

To conclude, the Gartner Hype Cycle narrowed the most promising research areas down to the broad fields of artificial intelligence, block chain, 5G, and transactive energy. At the same time, the Gartner Hype Cycle narrowed the main industrial successful applications down to the broad fields of connected home, smart cities, and IoT in oil and gas. Also, there is a peak of industrial interest in the subjects of deep learning, energy water nexus, IoT platforms and blockchain. This key information can represent a starting point when it comes to defining part of the content of an introductory energy informatics course.

## Teaching energy informatics

This section aims at discussing and illustrating the main features of an introductory energy informatics course, as well as potential variants that can lead to further more advanced/specialized energy informatics courses. This will be done in light of ongoing industry trends, forthcoming education needs, and the actual meaning of the energy informatics domain. It is important to highlight that the focus here is on discussing the content of a single energy informatics course rather than complete energy informatics study programmes. In contrast, a study programme is a continuous course of study comprised of courses and course groups. The length of the programmes depends on the degree to which they lead. Examples of study programmes can include: 1-year programmes, 3-year bachelor’s programmes, 5-year master’s degree programmes, 6-year programmes of professional study, 1–2 years master’s degree programmes that build on bachelor’s programmes and the like.

The objective of this section is to discuss the teaching of energy informatics as a single course within an existing study programme. Full energy informatics study programmes will be discussed in a separate paper.

From this point of view, the first question that arise is: which study programmes should or could include an energy informatics course? To answer this question it is important to bear in mind the key meaning and definition of the subject of energy informatics, which is using computer science to solve energy related problems. This requires a very strong applied focus and the ability to combine the fundamental disciplines of energy and informatics to address real world problems within the energy field. By keeping this in mind, it is possible to claim that an energy informatics course would find a suitable place in a variety of study programmes. In particular, suitable study programmes would be those that involve informatics related subjects, (such as computer science and the like), or energy and power related subjects, (such as electrical engineering and the like), or environmental related subjects, (such as environmental engineering and the like). In addition, suitable study programmes could be those applied study programmes where various applications to the real world are relevant, such as industrial engineering, physics or applied mathematics.

Another question that arises and that is worthy to discuss is whether an energy informatics course is suitable for a bachelor’s degree, or is it more suitable for a master’s degree? To answer this question it is important to bear in mind the interdisciplinarity that characterises an energy informatics course and the consequent need for such a course to put together competences in energy and informatics, as well as a broad knowledge of existing real world energy related issues. This means that basic competence and preliminary knowledge of fundamental energy and informatics concepts should be already part of the education background of the students. Therefore, an energy informatics course is more suitable as a specialisation course at the master’s level, where students have already built a foundation of fundamental subjects that they can utilise and further expand during the course itself. However, it is also important to bear in mind that the course will have to be adapted to the specific study programme where it is included. Indeed, students from different study programmes will have different backgrounds in fundamental subjects. Therefore it will be necessary to include some preliminary high level lectures where the necessary complementary skills in either energy or informatics will be refreshed and provided to the students, such that they will be able to comfortably follow the course.

### The energy informatics topics

The identification of a set of topics to build a common ground and foundation for an energy informatics course should never disregard the obvious fact that the subject “energy informatics” is basically made of two very well defined and self contained words: energy and informatics. Therefore, it is necessary to touch on and link both topics - energy and informatics - in order to provide a successful, meaningful and comprehensive energy informatics course.

The challenge here is that an energy informatics course given within different study programmes might tend to prioritise one aspect over the other, depending on the specific study programme’s philosophy. For instance, an energy informatics course given within a computer science study programmes may tend to overemphasize the computer science aspects and minimize energy related topics.

In order to be really successful, energy informatics teaching should cover both topics and not be limited to the computer science aspects or to the energy related aspects. Neither of the two aspects should be overemphasized nor underemphasized, but rather both should be linked, such that students will have the opportunity to use, develop, expand and gain skills on both sides.

To have a better understanding of what could and should fit within the two major topics of energy and informatics, it is worthwhile to look at both, current and future research directions, as well as current and future industrial interests/needs. The two previous sections briefly discussed research and industrial perspectives. Research wise, a wide variety of applications of computer science that address energy related problems have been identified, with a particular focus on the interdisciplinary approach that is needed to tackle such problems at different levels (technical, economic, social, legislative). Industry wise, as outlined in the previous section, the Gartner Hype Cycle found the most promising research areas are in the broad fields of artificial intelligence, blockchain, 5G, and transactive energy. The most successful industrial applications have been found within the broad fields of connected home, smart cities, and IoT in oil and gas.

Beyond the topics identified in the previous sections, it is also useful to look at what is covered by good quality scientific peer-reviewed journals that are specifically devoted to the two main areas of energy and informatics.

Relevant topics within the energy area can be identified by inspecting journals that address energy technologies, sustainable energy related issues, energy storage technologies, as well as power systems and electrical energy related topics. Good quality journals that address such topics are (but are not limited to): Journal of Energy (ISSN: 0360-5442), Journal of Cleaner Production (ISSN: 0959-6526), International Journal of Electrical Power and Energy Systems (ISSN: 0142-0615), Journal of Energy Storage (ISSN: 2352-152X), Journal of Electric Power Systems Research (ISSN: 0378-7796), Journal of Renewable Energy (ISSN: 0960-1481), IEEE Transactions on Smart Grid (ISSN: 1949-3053), IEEE Transactions on Industrial informatics (ISSN: 1941-0050). The subjects involved in such journals can be summarised as follows: heat transfer; energy conversion and efficiency; district heating and cooling; energy in buildings; solar thermal; geothermal and organic rankine cycle; thermodynamics; fossil fuels; biofuels; hydrogen; energy carrier; electricity demand; smart grids; smart energy and power systems; wind power; renewable energy; energy and transportation; integrated heating; cooling and electricity; national energy systems; energy and environment; energy planning; energy policy; energy economics; carbon capture; nuclear; energy storage technologies modelling, sizing, management, and analysis; storage integration within electric grids; business models for operation of storage systems and energy storage developments worldwide; generation, transmission, distribution and utilization of electrical energy; cleaner production and technical processes; sustainable development and consumption; policy for sustainability; renewable energy systems and components; alternative renewable energy solutions; and renewable and sustainable power generation.

Relevant topics in the informatics area can be easily identified by looking into the fundamental subjects that are generally taught within traditional computer science study programmes. What is normally found within such study programmes are the following fundamental disciplines: distributed systems, parallel programming, advanced database, programming, computers communication, cyber security, software engineering, green computing, artificial intelligence, operating systems, algorithms, big data analytics, and machine learning.

Given that the subjects of energy and informatics are very wide and broad, there is a need to select a subset of relevant topics to be part of an energy informatics course. How such topics should be selected is still an open question that should be addressed in two steps. First, it is important to define what is mandatory and needed as a basis for an energy informatics course. This can be referred to a common “fundamental framework” that would identify the energy informatics course. Secondly, it is necessary to identify “distinguishing features” that can be added on top of the fundamental framework, based on the lecturers’ research interests, the study programme’s main philosophy, the particular country’s needs, the job market needs in the particular area where the course is given, etc. Such distinguishing features are represented by those topics addressed during the course that differ from one department to another and from one university to another. They are called “distinguishing features” because they are supposed to reflect the specific relevant research interests and expertise that belongs to the specific lecturers that are giving the course. Of course, distinguishing features must be relevant for the overall energy informatics field.

### Fundamental framework of energy informatics courses

As outlined before, in order to provide a standard energy informatics university course, it is important to define a fundamental common framework and background that should be shared between the different universities. This means that it is important to agree upon what should be a mandatory foundation for an energy informatics course. When defining common topics that should be part of an energy informatics course, the following main assumptions should be considered:

- Energy informatics is a *broad* subject, which is neither about being a power/energy systems specialist, nor about being a computer science specialist. It is rather about building bridges between the areas.
- Energy informatics is an *interdisciplinary* subject, which means that the course should teach topics that link and put together the two areas of energy and informatics.

These two assumptions lead to the following main points to consider.

In order to address the instrinsic *broad* aspect of energy informatics, it is necessary to keep a high level approach to teaching. This means that it is not possible to go too deep into the details of energy/power systems or computer science within the course. But it is necessary to touch upon them and give a broad comprehensive high level introduction to them in order to provide a broad picture to students with different background and expertise. From this point of view, an energy informatics course should be accessible to students with different backgrounds from different study programmes (as outlined at the beginning of this section). This means that a broad high level introduction of the two areas of energy and informatics should be given within the course with particular regard to: energy and power systems fundamental concepts such as energy network, smart grids, microgrids, climate change, energy policy, energy economics, market mechanisms, smart buildings, future energy systems and low carbon systems transition, demand side management, storage technologies, electrical mobility; computer science fundamental concepts such as big data, data analytics, parallel and distributed computing basic concepts, platforms for data analyses, cyber physical systems, Internet of Things, cyber security and privacy protection, basic programming and modelling skills.

In order to address the *interdisciplinary* intrinsic aspect of energy informatics, it is necessary to provide lectures that teach the students how the knowledge in the two fields of energy and informatics can be linked. This can be done by discussing real world applications of ICT to energy related problems, where the two areas of energy and informatics complement each other. Examples of such applications can be represented by ICT for future energy systems; ICT for data analytics for smart energy systems, big energy data, platforms for data analysis; ICT for distributed generation and demand side management; ICT, modelling and simulation approaches for (multi-) energy networks and micro-grids; ICT, modelling and simulation approaches for energy-efficient mobility, charge management for electric vehicles, and smart grid integration of mobile storage; ICT, modelling and simulation applications for smart buildings, digital metering, occupant comfort, and user interaction; information systems for behavior change, and market mechanisms. Cross cutting issues can be included as well, by looking at applications of block-chain, cyber-security and privacy protection, interoperability, and verification of networked smart grid systems.

As the applications field is very wide, a selection of the most relevant and trendy topics should be made. This leads to the next section, where distinguishing features of an energy informatics course are discussed, in order to propose ways in which topic selection can be made wisely.

### Distinguishing features of an energy informatics course

The previous section identified a common framework and background for an energy informatics course; however, energy informatics is a very young and dynamic area, hence there should be some space for professors and lecturers to include specific relevant topics where their own imprint can also be visible. This means that, on top of the common framework and background, it is necessary to identify “distinguishing features” that will identify different universities and departments, depending on specific relevant available competences, research interests, as well as specific job market needs in the area where the course is given. An energy informatics course should follow a fundamental framework, but should also include some specific subjects that will identify different universities and departments, based on their stronger competences and research needs. Indeed, master courses are a way in which it is possible to recruit future PhD students and researchers; therefore, it is important that some space is reserved for the specific relevant subjects that are of interest to the lecturers such that the course will have a special imprint linked to the specific research that is run by the assigned lecturers.

Distinguishing features should refer to certain very specialised topics that are relevant for the energy informatics field. Examples of distinguishing features can be represented by (but are not limited to) a special focus on “Smart Energy and Power Systems Modelling” [68, 69] or “Green Computing” [70, 71], or “Big Energy Data Analytics” [72, 73] and “Machine Learning” [73, 74].

Indeed, computer science is not only about computers. Computer science is also about the clever use of limited resources. From this point of view, “Smart Energy and Power Systems Modelling” can play an important role in building bridges between energy and power systems problems and computer science aspects of modelling [75]. This refers to the process of building computer models of energy systems in order to analyze them. Models often use mathematical optimisation to minimise or maximise an objective function by fulfilling a set of equalities and inequalities in order to provide optimal decisions in terms of investment and/or operations. Mathematical modelling has strong potential for real-world applications in general [76]. Within the energy informatics domain, optimization models can be built to study different energy and power systems problems, focusing on different levels of details. Broad high level models of the whole European energy system [77] can be found in literature, as well as local models for smart microgrids design [14] or thermal networks design [78]. Different models will address problems by focusing on investment decision making or operational management of the systems. When operational optimization is the focus, more detailed optimal power flow models for network restructuring and reconfiguration [9], can be developed. However, when the focus changes and shifts towards higher level investment decision making, for instance with multihorizon multiyear perspectives [79], the network flow representation can be simplified, depending on the specific research questions to be addressed, It is possible to look into more detailed models for smaller systems like charging sites [38] or buildings [80], and further move down towards the detailed and specific models of single generator units [81] or specific storage technologies [82, 83]. By applying modelling and optimization to the specific field of power and energy systems, it is thereby possible to potentially touch all the topics listed in the Energy Journal subject classification, as long as the technical competences of the specific subjects are available.

Computer science is closely linked to modelling. Indeed, mathematical modelling requires the development of algorithms (exact algorithms, as well as heuristics or metaheuristics) that can be implemented using programming languages (e.g. Python and the related package Pyomo [84] and Julia and JuMP [85] are becoming very popular). The emphasis on algorithmic thinking is a common ground in computer science and operations research (where modelling and optimization represent the core). Moreover, cluster computing, together with parallel and distributed computing, can provide the computational requirements needed to solve bigger real world instances. Modelling also requires data management and manipulation, which can be achieved through machine learning. Indeed, while machine learning aims at extracting the knowledge from the data, the “Smart Energy and Power Systems Modelling” aims at converting such knowledge into optimal decisions.

“Green computing” can also represent a distinguishing feature of an energy informatics course. The objective of green computing is to investigate ways to make computing as a whole more sustainable, as well as ways to save energy in software and hardware systems. It is therefore a subtopic of energy informatics with a very specific focus on improving the efficiency and consumption of computing systems.

Finally, “Big Energy Data Analytics and Machine Learning” may also be given a special focus within an energy informatics course. They provide an energy perspective on Big Data. Indeed, the energy sector collects large amounts of data, on a continuous basis, both from the supply side and from the demand side. In smart grids, the main source of data is the advanced metering infrastructure, together with various intelligent devices such as sensors and thermostats, used throughout the whole process of power generation, transmission, distribution, and consumption. Other big data sources are weather data, mobile data, thermal sensing data, energy database, clean energy data, electric vehicle data, transmission line sensor, real estate data, dynamic pricing, and energy consumption control through behavioral analysis. From a Computer science perspective, this means addressing the challenges of database integration, data storage capacity, extraction of knowledge from data through machine learning, and security and privacy issues. Recent works and research directions addressing applications of big data and machine learning for energy and power related problems can be found in [86–88].

Beyond the specific examples mentioned above, any other similar topics that allow applications of computer science to specific energy related problems can represent a distinguishing feature that adds a special touch to an energy informatics course.

### Addressing interdisciplinary and cross cutting education needs

As discussed in the previous sections, energy informatics is a very broad and interdisciplinary domain. Therefore it is important to discuss how such interdisciplinarity should be addressed, both on the teachers side and on the students side. Two main questions arise that are worthy of being discussed:

- What is the ideal background of students following an energy informatics course?
- How should the didactic of teaching change when providing an interdisciplinarity course?

As for the first point, recommended prerequisites can be identified within fundamental subjects such as calculus, physics, basic programming skills, algorithms and data structure fundamentals. This fundamental background should prepare the students for picking up high level energy and power systems related topics, as well as computer science and mathematical methodologies to solve the related applied problems. As outlined in the previous sections, it is important to make the course accessible to a wide variety of students, therefore the course should be self-contained and provide suitable introductory material to the main topics that will be discussed.

The second point is related to the need for the didactic approach to respond to the interdisciplinary education needs. The teaching skills of lecturers should be further developed towards interdisciplinarity, and lecturers with an existing interdisciplinary background and research interests should be prioritised for an energy informatics course. In addition, a single lecturer might not be enough to handle a full energy informatics course and more lecturers should be involved due to the intrinsic interdisciplinarity. This means that guest lecturers from different academic disciplines with different backgrounds relevant to the course should be provided. This would add value and it would make the overall course structure more interesting for the students.

As discussed in [89] it is important to identify a good compromise between the generic approach and the more specialised approach that can be used within the course. Generic topics aimed at drawing the broad picture and framework can be covered at a more superficial level, as opposed to the more specialised topics that represent the distinguishing features outlined in the previous sections. The generic/specialist split can range from 50/50 all the way to 20/80 depending on the specific choices, available competences, research and industrial needs.

Another important issue to address is related to the need of keeping the energy informatics course at the research forefront, in order to constantly provide the students with the most novel topics within the field. In order to address such cross cutting education needs, the teaching material should ideally be represented by the latest and highest quality papers published in the scientific literature. Indeed, for such a novel and dynamic domain like energy informatics, reading material can become obsolete very quickly, and therefore it is necessary to constantly keep an eye on the most updated literature in order to provide the students with the most novel inputs. This way the teaching will always be at the forefront. In addition, guest lectures from industry outlining the most recent research and development that has led to successful real world applications will keep the course modern, fresh, and up to date. Industrial guest lectures should aim at preparing students to have enough knowledge about interdisciplinarity and to be able to apply the knowledge in their future jobs in industry.

### Experiences of energy informatics past and ongoing master level courses

Energy informatics as an initiative in research an education was launched in 2016 at University of Oslo’s Department of informatics, sponsored by UiO:Energy (which is the coordinating hub for energy research, education and outreach at the University of Oslo) and the energy company Equinor (former Statoil) through their “start-up” funding of an adjunct and a full professorship, respectively. Since 2017 an introductory course in energy informatics has been offered on a regular basis. The goal of the course is to lay the foundation for understanding how state-of-the-art ICT models, tools and techniques can be leveraged to create more sustainable energy systems, focusing on energy use in particular. The design of the course offers a fundamental framework. This means that the course is providing a broad overview of the most important topics that connect energy and informatics, and as such, provides an idea of what a fundamental framework of energy informatics is about.

The course is primarily designed with computer science students in mind, assuming some ICT knowledge and background in programming as a minimum. However, students with other backgrounds are also admitted to the course, although it is recognised that it might be more challenging for such students to pass the course, in particular with respect to mandatory assignments requiring programming. This is mitigated to some extent by formulating assignments that can be solved using libraries offered by programming platforms and languages such as Matlab, R and Python. These are platforms and languages that are increasingly being used in research and education in other departments and faculties across the University. Our experience is that this has worked quite well for the large majority of students.

Being a computer science department, the fundamental framework of our energy informatics course naturally focuses on some basics of power systems and smart grids, but it also reviews the most relevant ICT models, tools, and techniques applicable for addressing challenges in smart grids. The students also learn how cloud computing, cyber-security, big data, machine learning, game theory and optimization can be applied in smart grids with integrated solar and wind power, energy storage and electric vehicles. Topics addressed include machine learning for renewable energy forecasting, game theory for energy markets, blockchain technology in energy systems, demand response, energy neighbourhoods, EV charging, and green computing. Another important feature of the course is the use of guest lecturers from industry for every topic in the course. Our experience is that providing a per topic industrial perspective motivates the students in their course work through a better understanding of the relevance of the knowledge they are about to acquire in the course. This relevance is in solving societal challenges related to energy, as well as more clearly seeing later job opportunities.

As an emerging field, energy informatics is in its infancy at most universities and thus experiences limited teaching resources. Although we feel the energy informatics course outlined above as been successful, with an increasing interest from both students and industry, it is challenging to provide coverage of energy informatics with proper depth on all relevant topics without sufficient resources. As an immediate mitigation to this situation, one way forward could be to establish an energy informatics course exchange program between universities that have initiated activities in this field. This way, a number of energy informatics courses with different distinguishing features could be offered to the students at each university participating in the program. To this end, University of Oslo is currently developing a course with the working title “AI for Smart Energy”, while University of Tromsø is developing a course called “Smart Energy and Power Systems Modelling”. Both are to be included in a courses exchange program between the two institutions. A possible challenge is that this will require some level of coordination of course content. On the other hand, one thing we have gained from the COVID-19 pandemic is improved skills in providing distance lectures using digital tools; something a courses exchange program would definitely benefit from.

## Opportunities and challenges for interdisciplinary teams

Energy informatics brings forth experts from different disciplines to solve a single problem. While the objective of the project might be singular, different streams understand it differently and therefore answer differently. Therefore the key to managing differences is to agree on the ground rules of not only “what it is about”, but also “what it is not about”.

An interdisciplinary group (research or industrial) contains members with different expertise while having a streamlined and singular objective [90]. The authors present a framework for [91] discussing the challenges facing an inter-disciplinary research team. In [92] the authors present a road-map for successful interdisciplinary education research, proposing a reflective approach. The current research trends and projects often cover different departments while having a single objective. For instance, renewable energy covers departments such as material science, electrical power engineering, computer science, mechanical engineering, social sciences, etc. Indeed, an inter-disciplinary team has the opportunity to cover a wider spectrum of the problems and the advantage therefore is that the solution developed is applicable to multiple scenarios and circumstances. However, the challenge is that it takes considerably more time in comparison to the uni-dimensional research/industrial objectives. Having an interdisciplinary team is more relevant than ever since society is moving towards a cognitive and interconnected system. In the past finding a singular solution to a problem was relevant. Indeed, this process has the caveat that multiple solutions are developed for the same problem from different disciplines, therefore time and effort are not optimally utilized. Since the world has become more connected, there is a higher demand for universal, optimal, and time effective solutions, which means developing industrial tools or investigating a research question more often requires competences from different disciplines than in the past. For instance, the Paris agreement to combat climate change is not achievable unless the whole world unites to take serious measures. The solar energy field, for instance, benefits from experts from material science, electronics, and power system disciplines. The efficiency of a solar photovoltaic panel material composition is better addressed by the material science experts, while the efficient integration to the grid belongs to the power systems domain. Therefore, inter-disciplinary research and industrial innovation are becoming the “new normal”.

The primary challenges of an interdisciplinary group include, but are not limited to, communication barriers and methodology. In order to enhance cooperation in an interdisciplinary research group, new inter-disciplinary courses are needed. From this point of view, the energy informatics discipline would be fundamental to bridge the gap between the different disciplines.

In an interdisciplinary group there are different visions, as there are different domains covered. In order to integrate the visions, intersection points need to be established. The intersections can be in form of partially overlapping domain-specific objectives with clear boundaries. For instance, a technique to define “what the objective is not” will further narrow the research differences.

A challenge for interdisciplinary teams is also choosing the right portfolio of skills. Interdisciplinary, per-se, is not always beneficial, namely if the portfolio of expertise is wrongly created. Indeed, interdisciplinarity is good, as long as the disciplines involved make sense when connected to each other within the specific research filed that is targeted. For instance, a proper interdisciplinary team is one where people have different, but complementary skills. If people’s expertise, visions, and scientific interests are too disconnected and do not have anything in common with each other, the resulting team will be unsuccessful in the long run. Having different disciplines working together should not result in a portfolio of “islanded” researchers running in different directions. From this point of view, a proper leadership is essential to ensure cohesion within the team and a proper exploitation of different resources towards common visions and objectives.

## Conclusions

In this paper we have surveyed education opportunities and challenges of the novel energy informatics domain in light of the current trends and future directions of research and industrial innovation.

A main observation is that research needs to move from multi-disciplinary approaches towards an interdisciplinary approach, thereby synthesising the knowledge and methods from different disciplines. This will enable energy research and development to address problems at social, economic, technical, and legislative levels together. Energy informatics is key for achieving this as the field educates a new generation of experts that can take on the new interdisciplinary challenges of future integrated, sustainable energy systems.

Our main recommendations on the content of an (introductory) energy informatics course is to ensure there is an even balance between energy topics and informatics topics, and to design the course as two parts – one part consisting of a fundamental framework of mandatory topics, ensuring a common energy informatics knowledge basis across teaching institutions, and one part consisting of distinguishing features reflecting the particular expertise and interests of each institution offering the course. Additionally, we presented an overview of energy and informatics subjects from which the fundamental framework can be composed. Furthermore, we recommended the course be self-contained by providing suitable introductory material to the main topics and including the use of several (guest) lecturers from different relevant disciplines to ensure the interdisciplinary nature of the course.

Another recommendation on the content of an energy informatics course is to ensure that industrial needs are taken into account, together with the most recent research trends, such that the expertise of the students can be properly shaped according to the real world status both in industry and academia. For this purpose, we have discussed an adapted version of the hype cycles for the specific case of energy informatics, where different technologies are illustrated based on maturity, adoption, and social application. Reflecting on the hype cycle adapted for the energy informatics field can represent a starting point for identifying and discussing the fields of application in an introductory energy informatics course. The hype cycle should of course be reviewed after some years and updated such that the different technologies are positioned in the right phase base (innovation trigger, peak of inflated expectations, through of disillusionment, slope of enlightenment).

As energy informatics is in its infancy with limited teaching resources at most universities, we proposed this limitation be mitigated by establishing an energy informatics course exchange program between universities that have initiated activities in this field.

This paper also discussed the opportunities and challenges of interdisciplinary teams. Our main recommendations encompass the importance of establishing good communication between the members of the team, such that different visions and perspectives can be integrated within a common intersection point. Moreover, the importance of choosing the right portfolio of skills has been identified as a key for success, since interdisciplinary, per-se, is not always beneficial if the portfolio of expertise is wrongly created. From this point of view, a successful interdisciplinary team should comprise people with different yet complementary skills that share a common vision. Proper leadership is essential to achieve this goals.
